# Pyrite cloning: a single tube and programmed reaction cloning with restriction enzymes

**DOI:** 10.1186/s13007-018-0359-7

**Published:** 2018-10-17

**Authors:** Matthew D. Fischer, Emmanuel Mgboji, Zhongchi Liu

**Affiliations:** Department of Cell Biology and Molecular Genetics, University of Maryland, College Park, MD 20742 USA

**Keywords:** Pyrite, Cloning, Restriction digestion, Single tube, Programmed reaction

## Abstract

**Background:**

Insertion of engineered DNA fragments into bacterial vectors is the foundation of recombinant DNA technology, yet existing methods are still laborious, require many steps, depend on specific vector configuration, or require expensive reagents.

**Results:**

We have developed a method, called “Pyrite” cloning that combines the traditional restriction enzyme digestion and ligation reaction in a single tube and uses a programmed thermocycler reaction, allowing rapid and flexible cloning in a single tube. After the Pyrite reaction and transformation, approximately 50% colonies contain the expected insert, which can be easily and quickly determined by colony PCR or blue-white colony screening. We also demonstrated that Pyrite cloning can be applied for different cloning purposes.

**Conclusions:**

The Pyrite cloning method reported here is a single tube and programmed reaction cloning with restriction enzymes. Compared to other cloning methods, Pyrite cloning is flexible, inexpensive, simple, and highly efficient.

**Electronic supplementary material:**

The online version of this article (10.1186/s13007-018-0359-7) contains supplementary material, which is available to authorized users.

## Background

Insertion of a DNA fragment into a bacterial plasmid is the cornerstone of recombinant DNA technology and the foundation of modern biological research. Though traditional restriction enzyme digestion and ligation has long served as the original recombinant DNA method, other cloning methods have been developed to date, such as the Gibson assembly [4], Gateway system [6], and Golden Gate cloning [3]. While each method offers specific advantages, all of them have certain limitations. For instance, Gateway cloning requires expensive reagents and relies on specific DNA sequences for recognition by the proprietary Clonase enzymes. Golden Gate cloning requires type II restriction enzymes, which are relatively few in number. Further, it requires that the type II restriction enzyme recognition sites be engineered into both the vector and the insert fragment [3]. Unlike either of the aforementioned methods, Gibson assembly does not require previously engineered nucleotide sites. However, oligo primers used to generate Gibson assembly products can be relatively long (> 40 bp) and expensive. Due to the sequence-specific nature of the Gibson overlaps, these PCR products can typically be inserted into only one vector. The same limitation exists for Advanced QUick Assembly (AQUA) cloning, Ligation-Independent Cloning (LIC), and Sequence- and Ligation-Independent Cloning (SLIC), as all three methods depend on overlaps that often restrict destination vector selection to one option [1, 2, 5].

Traditional restriction enzyme-based cloning offers a vast selection of restriction enzymes with distinct recognition sites and vector choices, but this method suffers from relatively low efficiency and is generally more time consuming and laborious. Although certain one-step methods utilize restriction enzyme digestion and ligation, they require specific vector design and lack flexibility between systems [3, 9]. Improved restriction digestion-ligation (IRDL) cloning uses traditional type I restriction enzymes but requires the entry vector to contain the *ccdB* selection gene to eliminate cells containing the empty vector [9]. This limits the practicality of IRDL cloning only for vectors that have been constructed to contain this negative selection gene.

Here, we describe a modified procedure inspired by Golden Gate cloning and based on traditional restriction enzyme digestion and ligation. Because the multiple-step cloning procedure is condensed into a single tube and programmed incubation, this cloning method, dubbed “Pyrite cloning”, significantly reduces the labor and costs associated with the traditional restriction enzyme-based cloning. Additionally, it enables biologists to take advantage of the vast availability of traditional type I restriction enzymes and the myriad of pre-existing cloning vectors. The technique is easy, simple, applicable to different cloning purposes, and should greatly facilitate research advances.

## Methods

### Isolation of PCR products

All PCR products used in cloning reactions were amplified by NEB Q5 High Fidelity DNA polymerase using the following PCR program: 98 °C for 30 s, 30 total cycles of 98 °C for 5 s, 15 s at an annealing temperature based on primer sequence and calculation using the NEB Tm calculator (http://tmcalculator.neb.com/#!/main), and 72 °C for a variable amount of time depending on the amplicon size (10 s/kb). A final extension at 72 °C for 2 min concluded the reaction. Reagent volumes were as described in the Q5 High Fidelity DNA polymerase manual. 5 µl of PCR product were first run on a 1% agarose gel to determine efficiency of the amplification reaction. PCR products were purified using the Machery-Nagel “PCR clean-up Gel extraction” kit according to the manual and quantified using a Thermo Scientific Nandrop 2000c Spectrophotometer.

### Pyrite reaction

**Fig. 1:**
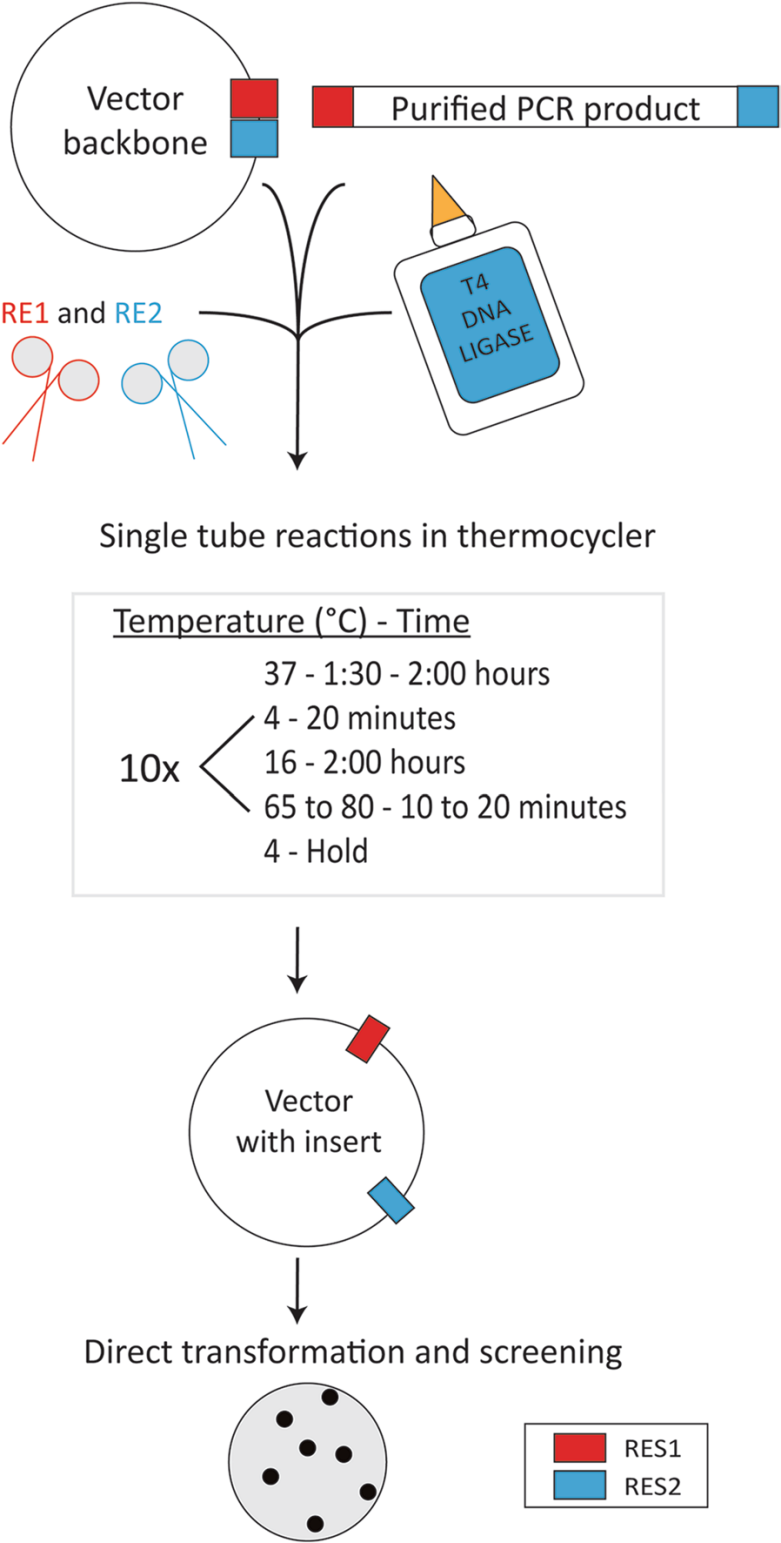
Schematic diagram of Pyrite cloning and results. Diagram of Pyrite cloning. An intact plasmid vector and a DNA fragment (purified PCR product) with compatible restriction enzyme sites (RES1 and RES2) are incubated in a single tube together with the restriction enzymes (RE1 and RE2) and T4 DNA ligase. After the Pyrite reaction (incubation condition shown in box), the reaction can be directly transformed into *E. coli* without purification. Colony PCR will then screen for those colonies containing vectors with inserts

**Fig. 2:**
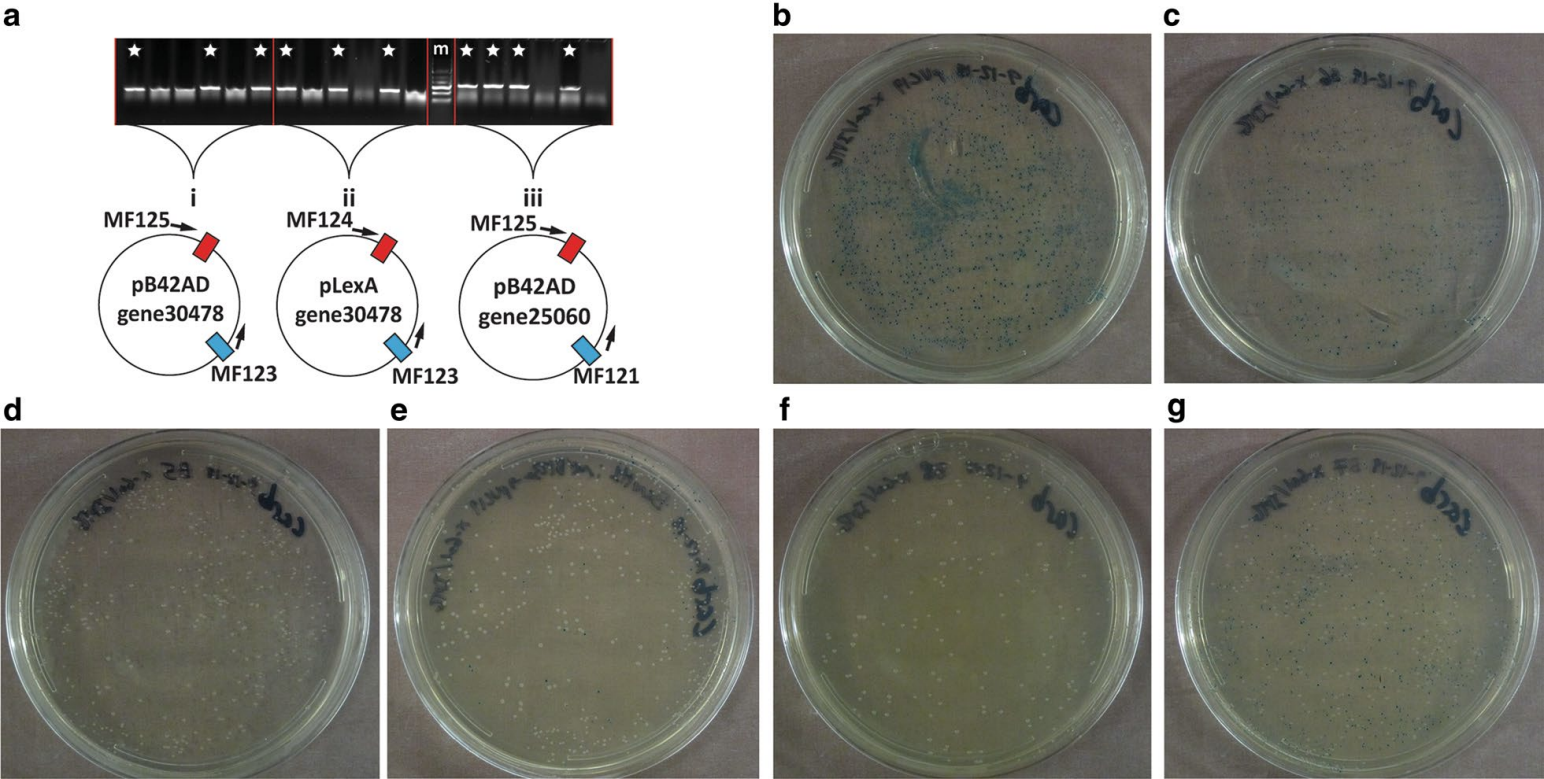
Determination of Pyrite cloning efficiency using colony PCR and blue-white screen. **a** Examples of colony PCR from three different reactions (i, ii, and iii), showing about 55% colonies (10/18; star) with the expected insert. “m” indicates marker lane (Goldbio 100 bp DNA ladder). Reaction i screened for insertion of *F. vesca* gene30478 CDS into pB42AD, reaction ii screened for insertion of *F. vesca* gene30478 CDS into pLexA, and reaction iii screened for insertion of *F. vesca* gene25060 CDS into pB42AD. **b**–**g** Blue-white colony screening used to determine the efficiency of the multiple applications of Pyrite cloning. Colonies are formed on antibiotic containing LB agar plates spread with X-gal and IPTG. **b** Control experiment showing 100% blue colonies after transformation of vector pUC19. **c** pUC19 was put into the Pyrite reaction to determine background re-ligation in the absence of DNA fragments. A smaller number of blue colonies was observed. **d** Pyrite reaction to insert *F. vesca* gene25060 CDS into pUC19. 100% white colonies were observed. **e** Pyrite cloning of gene25060 into pUC19 with SalI and BamHI-HF, a heat-resistant restriction enzyme, yielded 83% white colonies. **f** Simultaneous cloning of a *F. vesca* gene31413 into two vectors, pUC19 and pSanFran, in the same tube. The result of cloning gene31413 into pUC19 is shown. **g** Swapping eGFP from pSanFran to pUC19 with the Pyrite reaction, yielded 49.3% white colonies

The Pyrite cloning steps are outline in Fig. 1. Briefly, 2 μl of 10 × T4 DNA ligase buffer (or standard NEB enzyme buffer supplemented with 1 mM ATP), 400 units (1 μl) of NEB T4 DNA ligase, 6 units (0.3 μl) of each NEB restriction enzyme, 0.045 pmol of vector, 10 times (0.450 pmol) of purified insert fragment, and water to bring the final volume to 20 μl are mixed on ice. The reaction mix is then put into a preheated, programmed thermocycler to initiate the Pyrite reaction. The program (Fig. 1) consists of a first step of 37 °C for 1:30–2:00 h and then cycles between 4 °C for 20 min and 16 °C for 2:00 h. While it is possible that the ligated reaction products are redigested by the restriction enzymes during this step, the repeated cycling of these lower temperatures is likely to enrich the desired Pyrite reaction product. A final deactivation step at 65–80 °C is necessary to prevent digestion of the reaction products following removal from the thermocycler. This step should be included even if restriction enzymes that are resistant to heat inactivation (such as BamH1) are used. The product is then held at 4 °C or on ice. 1–2 μl of the reaction is used directly for transformation with electroporation competent 10β *E. coli*. For most experiments, High-Fidelity (HF) restriction enzymes were used. However, standard restriction enzymes such as XhoI (Fig. 2a) also worked in Pyrite cloning.

### Colony PCR screening of positive clones

Colony PCR was conducted to screen colonies that contain the vector with correct insert. Each colony PCR reaction has 10 µl volume: 1 µl of standard 10X Taq buffer, 0.17 µl of each primer (stock concentration of 10 µM), 0.17 µl of 10 mM dNTP, 0.25 units (0.05 µl) of Taq DNA polymerase, and 8.44 µl of water. Colonies were individually picked with sterile 10 µl pipet tips and dipped into the individual PCR test tubes. The reactions were put into a pre-heated thermocycler starting with 95 °C for 5 min and then 30 cycles of following: 95 °C for 30 s, 15 s at an annealing temperature based on the NEB Tm calculator (http://tmcalculator.neb.com/#!/main), and 68 °C for a specific time based on amplicon size (60 s/kb). Lastly, a final extension at 68 °C for 5 min concluded the reaction. The entire reaction was run on a 1% agarose gel to determine positive colonies. Six colonies are usually sufficient to identify at least one positive clone (Fig. 2a).

### White/blue colony screening of positive clones

Alternatively, the positive clones can be identified by the white/blue colony screen when one uses vectors containing lacZ such as *pUC19* (Fig. 2b–g). 50 µl sterile water, 40 µl of 20 mg/ml X-gal in dimethylformamide, and 5 µl of 0.8 M IPTG solution were applied on top of an antibiotic plate and spread until dry. Transformed bacteria were concentrated by centrifugation, resuspended in 200 µl LB, and all were plated and incubated at 37 degrees Celsius for 18 h. Blue colonies indicated negative cloning events while white colonies indicated positive cloning events due to disruption of the *lacZ* gene coding for the β-galactosidase within the pUC19 vector [8].

### pSanFran and pMerlin vector engineering

pSanFran was engineered from the pCR™8/*GW*/*TOPO*^®^ vector. pCR™8/*GW*/*TOPO*^®^ was amplified with MF149 and MF150 to generate 5′ EcoRI and 3′ SalI cutsites around the insertion site in addition to AarI cutsites for downstream Golden Gate cloning. GUS 3′ UTR was amplified from pMDC162 with MF151 and MF152. The resulting PCR fragments were ligated with Gibson Assembly and transformed into *E. coli*, generating the pSanFran clones (Additional file 1: Figure S1A).

pMerlin was engineered from the pMOD_C0000 vector. pMOD_C0000 was amplified with MF153 and 154 to generate 5′ EcoRI and 3′ SalI cutsites around the insertion site in addition to AarI cutsites for downstream Golden Gate cloning. The *Tobacco etch virus* leader sequence was amplified with MF155 and 156. The resulting PCR fragments were ligated with Gibson assembly and transformed into *E. coli*, generating the pMerlin clones (Additional file 1: Figure S1B).

## Results

### The Pyrite cloning reaction

Figure 1 illustrates how the Pyrite cloning condenses traditional, multiple-step cloning by combining the digestion and ligation reactions into one test tube subjected to programmed thermocycling. Pyrite cloning uses a single buffer and does not require purification or isolation of digested vector prior to ligation. Both restriction digestion and ligation processes are completed in a single tube with the use of a programmed thermocycler (Fig. 1). The principle of the protocol is that the T4 DNA ligase and certain NEB restriction enzymes, such as the High-Fidelity (HF) series that are compatible in the Cutsmart buffer, can function in the T4 DNA Ligase buffer. However, standard NEB restriction enzymes such as XhoI have been shown to function in Pyrite cloning, so other standard NEB restriction enzymes with high functionality in the Cutsmart buffer may function in Pyrite cloning (Fig. 1). Additionally, the T4 DNA ligase and DNA restriction enzymes appear to be optimal at different temperatures. Therefore, the kinetics and thermodynamics of the digestion and ligation reactions in addition to sticky-end annealing or blunt-end ligation can be manipulated to favor digestion in the first thermocycler step (37 °C for approximately 1.5 h) and ligation in the next step, that cycles between 4 and 16 °C (for 10 cycles) (Fig. 1). This temperature cycle ligation facilitates annealing of DNA ends at 4 °C and then ligation at 16 °C [7]. A final deactivation step at 60–80 °C prevents digestion of the reaction products. As a result, Pyrite cloning requires minimal preparation for inserting one fragment into a vector.

1–2 μl of the resulting reaction is transformed into *E. coli*, which are grown on LB medium containing appropriate antibiotics. Colony PCR is conducted to screen for colonies containing the vector with insert. An alternative way to screen for successful insertion is to use the blue/white screening to take advantage of vectors such as pUC19 containing a functional lacZ gene coding the β-galactosidase. Successful insertion disrupts the lacZ gene and leads to white colonies when the transformed cells are plated on LB plates containing X-gal and IPTG as well as appropriate antibiotics. Colonies containing plasmids without the insert will be blue in color. This second approach also provides a “global” view of the insertional frequency based on while to blue colony ratio.

### Pyrite reaction can efficiently insert a purified PCR product into a vector

Pyrite cloning was used to insert two wild strawberry (*Fragaria vesca*; *F. vesca*) coding sequences (CDS), gene25060 and gene30478, into two plasmids in independent experiments (Fig. 2a). The *F. vesca* CDS was amplified with NEB Q5 High Fidelity polymerase. Forward and reverse PCR primers were designed to introduce 5′ EcoRI and 3′ XhoI recognition sites, respectively, along with four additional nucleotides at the 5′ to ensure optimal digestion efficiency (Additional file 1: Table S1). The resultant PCR products were first purified with the Machery-Nagel “PCR clean-up Gel extraction” kit according to the user manual. Purified PCR products were then put into Pyrite cloning reaction for insertion into either the pB42AD (also known as pJG4-5) or pLexA (also known as pEG202) vectors. Afterwards, the Pyrite cloning reaction was transformed into *E. coli* and spread on carbenicillin plates. Colony PCR screening, using a vector-specific forward primer and an insert specific reverse primer was conducted on six colonies for each Pyrite reaction (Fig. 2a). In each case, 3–4 positive colonies were identified among the 6 colonies screened, suggesting that approximately 50% of the colonies contain the insert (Fig. 2a). Therefore, colony PCR is recommended as means to quickly identify positive clones.

To get more accurate Pyrite cloning efficiency, the blue/white colony screen method was used with the vector pUC19, which contains a lacZ gene that is disrupted by insertion [8]. First, *E. coli* colonies transformed with intact pUC19 plasmid were all blue on LB agar plate supplemented with X-gal, IPTG, and carbenicillin (Fig. 2b). To determine background self-ligation, pUC19 was put into the Pyrite reaction with EcoRI-HF and SalI-HF in the absence of DNA insert fragments. Blue colonies were formed but at significantly lower number than the intact pUC19 control (Fig. 2c), suggesting that a portion of the empty vector may religate while others may not during the Pyrite reaction. Next, the Pyrite reaction was used to insert the *F. vesca* gene25060 CDS DNA fragment into pUC19. The pmols of pUC19 vector and the gene25060 fragment in the reaction were calculated from purified DNA in a 1:10 vector: insert ratio, which yielded 100% white colonies (Fig. 2d). Taken together, the X-gal based while/blue screening suggests that the efficiency of the Pyrite reaction is consistently high (> 90%; Table 1) when there is a high ratio of insert: vector.

**Table 1:** Percentage of blue and white *E. coli* colonies resulting from various Pyrite cloning applications shown in Fig. 2

Application type	Figure 2	Blue colonies	Total colonies	Percent blue	Percent white
Background control	C	> 100	> 100	100	0
Insertion	D	0	> 100	0	100
Use of BamHI	E	78	458	17.0	83.0
Simultaneous cloning (pUC19)	F	0	202	0	100
Simultaneous cloning (pSanFran)	n/a	n/a	401	n/a	100
Vector swap	G	319	629	50.7	49.3

### Heat-resistant restriction enzymes can be used in Pyrite reaction

The above examples used restriction enzymes that could be heat inactivated, and this deactivation could be critical to prevent redigestion and hence the integrity of the final product. To test if Pyrite cloning could be conducted with enzymes that could not be inactivated by heat, BamHI-HF and SalI-HF were used to insert *F. vesca* gene25060 CDS into pUC19 (Fig. 2e). While SalI can be deactivated, BamHI lacks this quality. After Pyrite cloning, 83.0% colonies were white (Table 1; Fig. 2e). This suggests that even heat-resistant restriction enzymes can be used in the Pyrite reaction, providing additional flexibility.

### Pyrite cloning is flexible and suitable for several cloning applications

**Fig. 3:**
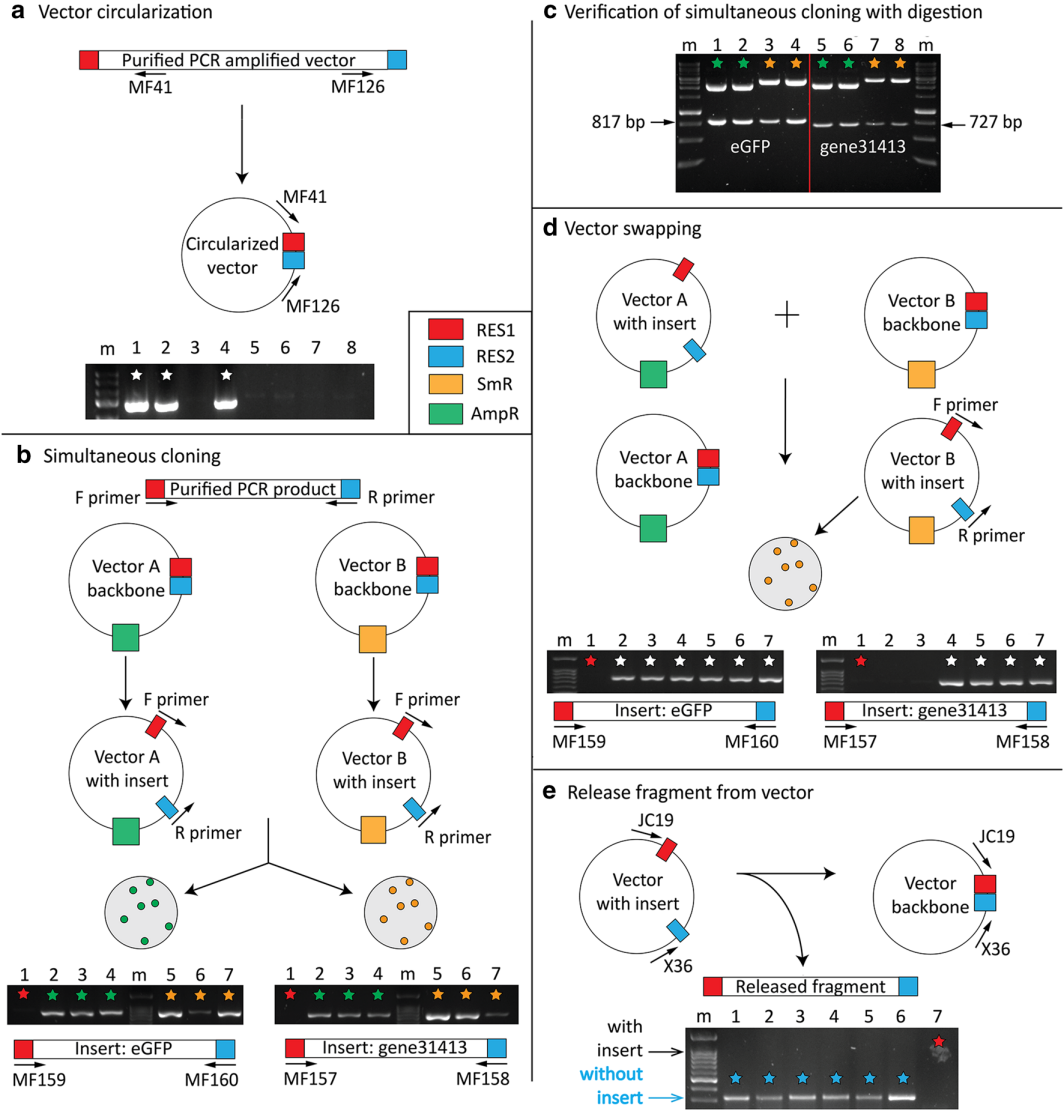
Multiple applications of Pyrite cloning. **a** Circularization/ligation of a linear DNA fragment. A PCR amplified linear vector can be cut and ligated using the Pyrite reaction. pLacZi was amplified with primers MF143 and MF144 and put in the Pyrite reaction with HindIII-HF, restoring the HindIII site in pLacZi. MF41 and MF126 are PCR primers used to test successful cloning. Three of the eight colonies tested were positive and marked by white stars. **b** Insertion of a DNA fragment into multiple destination vectors in a single reaction. The transformed cells can be plated on different antibiotic containing media to select for the desired vector. The gel image on the left shows the insertion of eGFP into two vectors simultaneously; the image on the right shows the insertion of gene31413 CDS into two vectors simultaneously. Red star indicates negative control (no template). Green stars indicate the vector pMerlin, which confers AmpR. Orange stars indicate the vector pSanFran, which confers SmR. “m” indicates marker lane (Goldbio 100 bp DNA ladder). **c** Restriction digestion of extracted plasmid DNA by EcoRI and SalI to release the cloned fragment. Proper insert and vector size confirmed colony PCR results. Arrows indicates the released insert of 817 bp and 727 bp respectively. Green star indicates vector that conferred AmpR (pMerlin). Orange star indicates vector that conferred SpecR (pSanfran). m indicates Goldbio 1 kb DNA ladder. **d** Insert swapping between vectors with different antibiotic resistances. Two successful examples are shown with 67–100% efficiency (positive PCR products marked by white stars). **e** A vector with an insert can revert to the empty vector by releasing the insert with the Pyrite reaction. Gene31413 CDS was removed from the pCR™8/*GW*/*TOPO*^®^ vector with EcoRI-HF. All colonies screened contain the empty vector without an insert (blue stars)

We tested several other utilities of the Pyrite cloning (Fig. 3). In addition to effectively inserting a purified PCR product into a vector (Fig. 2a, d, e), the Pyrite reaction can circularize a vector that has been amplified by PCR (Fig. 3a). As shown, pLacZi was amplified with MF143 and MF144 and put into the Pyrite reaction with HindIII-HF, restoring a Hind III site in pLacZi. This application can be used to engineer modifications into a vector, such as altering the multiple cloning site by incorporating new enzyme recognition sites in the PCR primer.

In addition, a single Pyrite cloning reaction may be utilized to insert a fragment simultaneously into two different vectors containing different antibiotic resistance genes (Fig. 3b). In this case, clones containing the desired vector could be selected with the corresponding antibiotic. Such a reaction would save time and reduce costs, as fewer reagents would be consumed in generating two different constructs. Tests with two different inserts, eGFP (Fig. 3b left gel) and the CDS of *F. vesca* gene31413 (Fig. 3b right gel) have respectively shown 100% efficiency with vectors pSanFran and pMerlin as determined by colony PCR (Fig. 3b). The resulting positive colonies were further confirmed by plasmid extraction and diagnostic restriction digests (Fig. 3c). To test blue/white screening, *F. vesca* gene31413 CDS was cloned into two vectors simultaneously, pUC19 (Fig. 2f; Table 1) and pSanFran (Table 1), and both yielded 100% white colonies.

Similarly, Pyrite cloning can swap DNA fragments between vectors containing different antibiotic genes (Fig. 3d). If the vectors have the same antibiotic resistance, the undesired vector may be cut with a restriction enzyme that only recognizes the undesired vector before transformation into *E. coli*. This particular application facilitates downstream application of an entry clone, much in the same way that Gateway recombination-based cloning facilitates swapping between entry and destination vectors, except that the Pyrite reaction requires neither Clonase nor destination vectors with the ccdB gene. As shown in Fig. 3d, the colony PCR revealed that the efficiencies in two different cloning experiments swapping either eGFP or gene31413 from pMerlin to pSanFran are 100% and 67%, respectively. We also used the blue/white screen to test the efficiency of swapping eGFP from the pSanFran vector to pUC19 in a 1:1 pmol ratio with the Pyrite reaction, which yielded 48.3% white colonies (Fig. 2g; Table 1).

Finally, Pyrite cloning facilitates removal of a DNA fragment from a vector (Fig. 3e). For example, a previously cloned vector can be put into the reaction to liberate a fragment flanked with a restriction enzyme site (or two different enzyme cutting sites with same sticky ends). The resulting vector could self-ligate to recreate the empty vector, which could be easily detected with colony PCR. As shown in Fig. 3e, 100% of the colonies contain the vector without the insert when the pCR™8/*GW*/*TOPO*^®^ vector containing gene31413 was put into the Pyrite reaction with EcoRI-HF.

## Discussion

Though many recombinant DNA cloning methods have been developed over the years, traditional restriction enzyme digestion and ligation is still utilized due to its relatively low cost and flexibility. The Pyrite cloning reported here further improves this restriction enzyme-based cloning method, simplifying it into a single tube reaction in a programmed thermocycler, saving time, labor, and reagents as detailed in Table 2. While traditional restriction enzyme cloning takes 6 steps and 2–4 h laboring time, the Pyrite cloning consists of only 2 steps and takes 0.5–1 h labor. Compared with Gibson cloning, which requires typically 40 bp oligo primers costing about $20 per pair of primers, the Pyrite primers are typically 20 bp, costing ~ $10 per pair of primers. These advantages of Pyrite should significantly facilitate research progress in small or large institutions. Furthermore, Pyrite cloning only requires a vector with a multiple cloning site, necessitating minimal vector engineering and enabling recombinant DNA with essentially any vector. While Pyrite cloning has an inherent risk of generating empty vector and negative clones, colony PCR and blue-white screen demonstrated here indicate that multiple positives can be identified by screening so few as six colonies with colony PCR (Fig. 2a) or by a visual screen such as the white/blue screen with pUC19.

**Table 2:** Comparison of time investments to complete traditional cloning versus Pyrite cloning

Step	Traditional cloning	Time investment	Pyrite cloning	Time investment
1a	Digest vector	1–24 h	Prepare and incubate reaction	18–25 h
1b	Digest insert(s)	1–24 h		
2	Gel electrophoresis	15–45 min	Transform	1–2 h
3	Gel purification	1–2 h		
4	Ligation reaction	16 h		
5	Deactivate ligase	10 min		
6	Transform	1–2 h		
Total steps	6		2	
Total labor time	2–4 h		0.5–1 h	
Total incubation time	19–43 h		19–26 h	

Golden Gate cloning has demonstrated that type II restriction enzymes and DNA ligases could function in a single tube reaction. The cloning reaction described here, dubbed Pyrite or “Fool’s Gold” cloning, introduces a procedure in which the final product may be cut as it is formed due to the use of type I restriction enzymes. While this may seem to be inherently risky, the results demonstrate that it is an efficient means by which to conduct cloning. We do not know what contributed to the colonies that contain no insert. They are likely caused by either incomplete digestion or re-ligation of the vector. The large amount of insert DNA in the Pyrite reaction may stoichiometrically inhibit digestion of the vector, though this was not experimentally determined.

Following are a list of notes and considerations that may assist others with an interest in using Pyrite cloning:

1. Primers designed to introduce terminal restriction sites should include four additional nucleotides 5′ to the enzyme site (see Additional file 1: Table S1 and NEB specifications (https://www.neb.com/tools-and-resources/usage-guidelines/cleavage-close-to-the-end-of-dna-fragments and https://www.neb.com/-/media/nebus/files/chart-image/cleavage_olignucleotides_old.pdf?la=en).
2. 10:1 insert:vector pmol ratio is highly recommended to maximize positive cloning events. However, for insert swapping application (Fig. 3d), a 1:1 ratio is recommended.
3. PCR products should be purified to remove the Q5 High Fidelity DNA polymerase. In the absence of the purification, the restriction digested PCR products were found to be blunt ended and ligated into the vector in random orientation, limiting successful cloning events. It is likely that the Q5 High Fidelity DNA polymerase may function in the Pyrite reaction to fill in the sticky-ends. Thus, a purification step after the PCR is highly recommended prior to the Pyrite reaction.
4. Standard T4 DNA ligase is sufficient for Pyrite cloning.
5. In general, High-Fidelity (HF) restriction enzymes are recommended. Standard restriction enzymes are sufficient for Pyrite cloning, but they should be tested for functionality and fidelity in the T4 DNA ligase buffer. XhoI has been verified to work as shown in Fig. 2a, so other enzymes with full functionality in Cutsmart buffer may also work well for Pyrite cloning.
6. Any NEB restriction enzyme buffer may be used instead of T4 DNA ligase buffer in the Pyrite reaction so long as it is supplemented with 1 mM ATP.
7. It is suggested that the ligation cycling steps between 4 and 16 °C run overnight. However, 10 cycles are not necessary; eight cycles have been sufficient. Cycling less than 8 times may be effective but has not been tested.
8. Colony PCR screening or visual screening (either with LacZ or GFP) is necessary to identify positive clones, as some colonies will contain empty vector.
9. Insert and vector sequence should be carefully taken into consideration to avoid selecting restriction enzymes that could digest the sequence of interest.
10. While Alkaline Phosphatase, Calf Intestinal (CIP) has been utilized in traditional cloning to prevent vector self-ligation, it should be omitted from the Pyrite reaction as this is a one-tube reaction; the CIP will render both the vector and the insert incapable of ligating into the vector.
11. Heat-resistant restriction enzymes can be used in Pyrite cloning. In these events, the deactivation step (Fig. 1) should still be included in the program.

## Conclusion

In summary, the Pyrite cloning strategy described here simplifies traditional restriction enzyme cloning methods by combining the restriction enzyme digestion and ligation reactions in a single tube without the need to purify in between steps. It can greatly facilitate research in labs with limited resources as well as facilitate research experiences of high school or undergraduate students.

## Additional file

**Additional file 1.** **Figure S1.** pSanFran and pMerlin vector map. **Table S1.** Oligo primer sequences used in this study.
