# Supplementary material for: Pyrite cloning: a single tube and programmed reaction cloning with restriction enzymes
Source: Plant Methods. 2018 Oct 17;14:91. doi: 10.1186/s13007-018-0359-7 (PMC6192209; doi:10.1186/s13007-018-0359-7)
Supplement: Supplementary file 1 — Additional file 1. Figure S1. pSanFran and pMerlin vector map. Table S1. Oligo primer sequences used in this study. [file 13007_2018_359_MOESM1_ESM.docx]

**Supplemental Materials**

**Pyrite cloning: a single tube and programmed reaction cloning with restriction enzymes**

Matthew D. Fischer, Emmanuel Mgboji, and Zhongchi Liu*

Dept. of Cell Biology and Molecular Genetics

University of Maryland

College Park, MD 20742

*Corresponding author

Zhongchi Liu

zliu@umd.edu

**
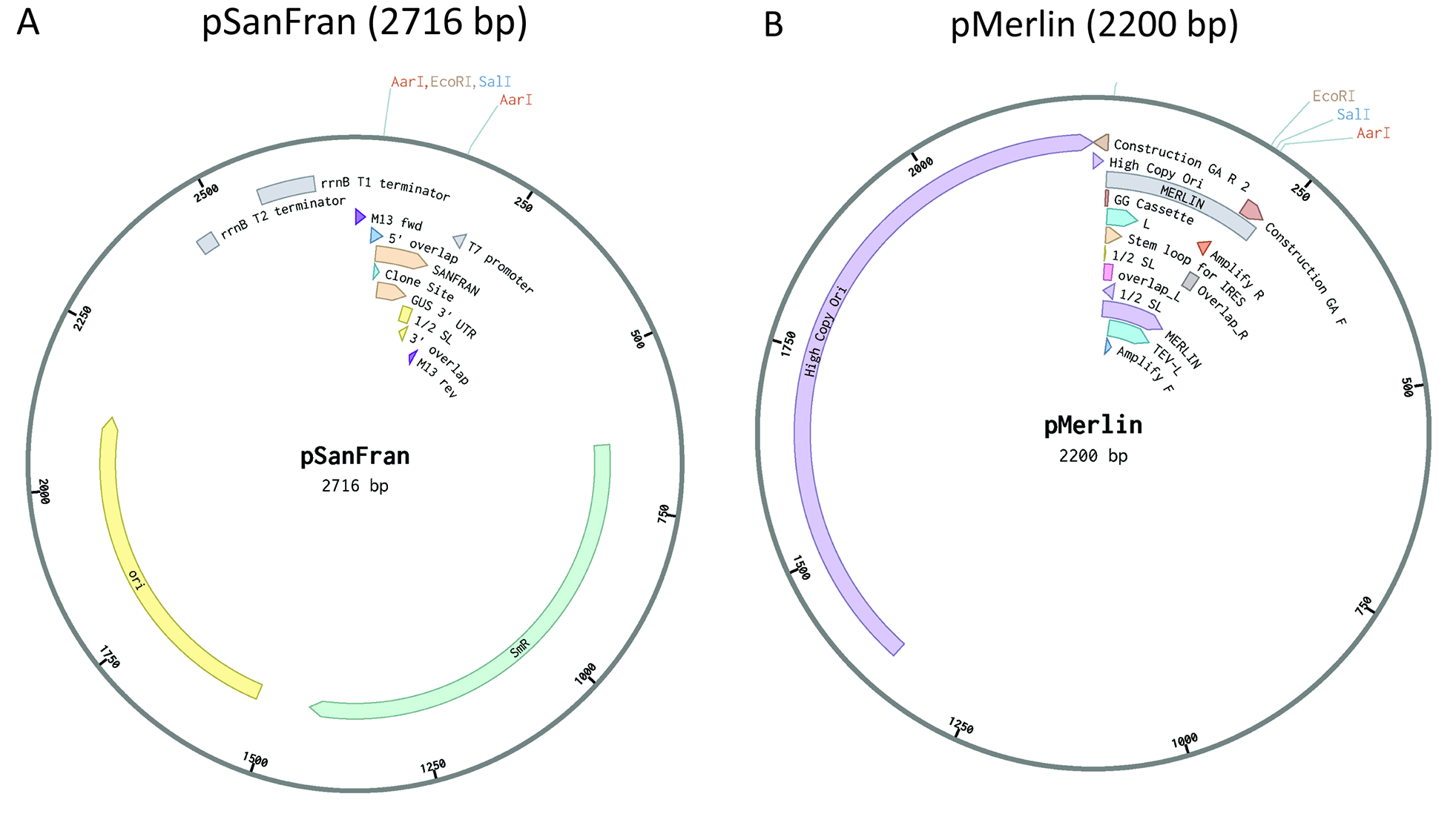
**

**Additional file 1: Figure S1: pSanFran and pMerlin vector map**

Both pSanFran (A) and pMerlin (B) serve as entry vectors for downstream Golden Gate cloning. Fragments can be inserted into both vectors using EcoRI and SalI. pSanFran was engineered from the pCR™8/GW/TOPO® vector. pMerlin was engineered from the pMOD_C0000 vector. See methods for engineering specifications.

**Table S1**: **Oligo primer sequences used in this study.**

| Primer | Sequence | Enzyme |
| --- | --- | --- |
| MF120 | 5’ **CCCG**GAATTCATGAATACAGGAGCTTGG 3’ | EcoRI |
| MF121 | 5’ **CCCG**CTCGAGTCAATCAACATCAGTTTCAAG 3’ | XhoI (Fig. 2A) |
| MF122 | 5’ **CCCG**GAATTCATGTCTAGCGGAAGGTC 3’ | EcoRI |
| MF123 | 5’ **CCCG**CTCGAGCTACTGCATAATCAAGCTCCT 3’ | XhoI (Fig. 2A) |
| MF124 | 5’ CGTCAGCAGAGCTTCACC 3’ | N/A; colony PCR primer for pLexA (Fig. 2A) |
| MF125 | 5’ CCAGCCTCTTGCTGAGTG 3’ | N/A; colony PCR primer for pB42AD (Fig. 2A) |
| MF126 | 5’ TGCCTCGAGGTCGACAGA 3’ | N/A; colony PCR primer for pLacZi (Fig. 3A) |
| MF41 | 5’ GGAATAAGGGCGACACGGAAATG 3’ | N/A; colony PCR primer for pLacZi (Fig. 3A) |
| MF143 | 5’ **GGGC**AAGCTTTTCAATTCATCATT 3’ | HindIII (Fig. 3A) |
| MF144 | 5’ **GGGC**AAGCTTGAATTCGAGCTCGGTACCCG 3’ | HindIII (Fig. 3A) |
| MF149 | 5’ CGACGAATTCGATCAAAAGCAGGTGCGGAGCCTGCTTTTTTGT 3’ | N/A; pSanFran engineering |
| MF150 | 5’ CAGATCTACGCGTACGTAAAAGCAGGTGCGACCCAGCTTTCTTGTA 3’ | N/A; pSanFran engineering |
| MF151 | 5’ CTGCTTTTGATCGAATTCGTCGACTGAATCAACAACTCTCCTGGC 3’ | N/A; pSanFran engineering |
| MF152 | 5’ TTTACGTACGCGTAGATCTGGGCCCAAGCTTGGGGAAATTCGAGCTCGG 3’ | N/A; pSanfran engineering |
| MF153 | 5’ CCCAGATCTACGCGTACGTTTTTGCAGGTGGGCGCTCTTCC 3’ | N/A; pMerlin engineering |
| MF154 | 5’ AGCAGAATTCGTCGACAGTGACTAGCAGGTGATGAAAAGCCTTAGG 3’ | N/A; pMerlin engineering |
| MF155 | 5’ CGTACGCGTAGATCTGGGCCCAAGCTTAAATAACAAATCTCAACACAAC 3’ | N/A; pMerlin engineering |
| MF156 | 5’ CACTGTCGACGAATTCTGCTATCGTTCGTAAATG 3’ | N/A; pMerlin engineering |
| MF157 | 5’ **CCG**GAATTCATGGAGGGTTATTTCGGTGT 3’ | EcoRI (Fig. 3B, D) |
| MF158 | 5’ **ACGC**GTCGACTCATACGTAGGAGATGTTGACTAGATC 3’ | SalI (Fig. 3B, D) |
| MF159 | 5’ **CCG**GAATTCATGGTGAAGACTAATCTTTTTCTC 3’ | EcoRI (Fig. 3B, D) |
| MF160 | 5’ **ACGC**GTCGACTTACAGCTCGTCCTTCTTG 3’ | SalI (Fig. 3B, D) |
| MF178 | 5’ **CGC**GGATCCATGAATACAGGAGCTTGG 3’ | BamH1 |
| MF179 | 5’ **ACGC**GTCGACTCAATCAACATCAGTTTCAAG 3’ | SalI |
| JC19 | 5’ GTTGTAAAACGACGGCCAGT 3’ | N/A; colony PCR primer for pCR8/GW/TOPO (Fig. 3E) |
| X36 | 5’ AGATTTTGAGACACGGGCCAG 3’ | N/A; colony PCR primer for pCR8/GW/TOPO (Fig. 3E) |

Note: Restriction enzyme sites are underlined. The sequence downstream of the restriction enzyme site belongs to *F. vesca* CDS of interest.
